# Long-term use of glucocorticoids for polymyalgia rheumatica: follow-up of the PMR Cohort Study

**DOI:** 10.1093/rap/rkac034

**Published:** 2022-05-11

**Authors:** Sara Muller, Samantha L Hider, Balamrit Singh Sokhal, Sarah A Lawton, Toby Helliwell, Christian D Mallen

**Affiliations:** 1 Primary Care Centre, Versus Arthritis, School of Medicine, Keele University, Keele; 2 Haywood Academic Rheumatology Centre, Midlands Partnership NHS Foundation Trust, Stoke-on-Trent; 3 School of Medicine; 4 Keele Clinical Trials Unit, School of Medicine, Keele University, Keele; 5 Midlands Partnership NHS Foundation Trust, Stoke-on-Trent, UK

**Keywords:** PMR, cohort studies, primary health care

## Abstract

**Objectives:**

PMR is a common inflammatory condition in older adults, characterized by bilateral hip and shoulder pain and stiffness. Reducing oral glucocorticoids, classically used for ≤2 years, are the mainstay of treatment. This study considers the factors early in the disease course that might be associated with prolonged treatment.

**Methods:**

Six hundred and fifty-two people with incident PMR were recruited from English general practices (2012–2014). Participants completed seven questionnaires over 2 years (used to allocate people to pain–stiffness trajectories) and a further long-term follow-up (LTFU) questionnaire a median of 5.16 years after diagnosis. Characteristics of those still taking and having ceased glucocorticoids were described and compared using Kruskal–Wallis and χ^2^ and Student’s 2-sample *t*-tests as appropriate.

**Results:**

Of the 197 people completing the LTFUQ questionnaire, 179 people reported ever having taken glucocorticoids. Of these, 40.1% were still on treatment, with a median (quartile 1, quartile 3) daily dose of 5 (1.5, 9)  mg. People still taking glucocorticoids were more likely to be older (72.5 *vs* 70.2 years, *P* = 0.035), live alone (31.8 *vs* 15.0%, *P* = 0.01) and have self-managed their glucocorticoid dose (39.1 *vs* 11.0%, *P* < 0.0001). They were also more likely to belong to a pain–stiffness trajectory class with sustained symptoms.

**Conclusions:**

PMR is not always a time-limited condition. Few patient characteristics are associated with prolonged treatment early in the disease course, but those who are older and who have sustained symptoms might be at greater risk. Although accurate prediction is not yet possible, clinicians should monitor people with PMR carefully to manage symptoms and reduce the cumulative glucocorticoid dose.

Key messages

- PMR is usually treated with oral glucocorticoids tapering over 2 years.
- We show that 40% of PMR patients are still treated after a median of 5.16 years.
- No predictors of extended PMR treatment were identified; therefore, all patients should be monitored carefully.

## Introduction

PMR is the commonest inflammatory rheumatic disease in older adults. It causes severe pain and stiffness in the hip and shoulder girdles and is usually accompanied by an acute phase inflammatory response. As such, PMR can cause significant disability [1] and severely impact on quality of life [1, 2].

A gradually reducing regimen of oral glucocorticoids is usually used to treat PMR and provides rapid relief from symptoms. Clinical guidelines recommend a starting dose of 12.5–25 mg of prednisolone daily, tapered to a stop over 18–24 months [3, 4]. There are concerns from professionals and patients regarding the potential side-effects of such long-term glucocorticoid treatment [1, 5–7], and evidence is emerging that people with PMR experience a high burden of incident glucocorticoid-related morbidities, including vascular (23% increased risk), respiratory (25% increased risk), endocrine (41% increased risk) and gastroenterological (21% increased risk) morbidities. Glucocorticoid treatment can also affect the eyes (37% increased risk), in addition to bone health (111% increased fragility fracture risk), and can cause or exacerbate mood problems (29% increased risk) or, in some cases, lead to psychosis in psychiatric conditions [8, 9].

There is also growing evidence that for some people with PMR, treatment is more protracted than guidelines suggest [10, 11]. A recent systematic review and meta-analysis [12] has estimated that 51% (95% CI 41, 61%) of people were still taking glucocorticoid treatment 2 years after diagnosis and 25% (95% CI 15, 36%) after 5 years and that symptoms do not necessarily remain well controlled during glucocorticoid treatment. The reasons for the heterogeneity in treatment and symptom course remain poorly understood, although it is plausible that they could be related to the initial presentation of PMR, the co-morbidity profile of an individual, which may change over time, or to the symptom tail [13]. The glucocorticoids (CS) legacy has been described as the long-term impact of glucocorticoid treatment, after the resolution of the PMR itself, including, for example, weight gain, hair loss, fragility fracture and diabetes. A symptom tail may relate to co-morbidities present before diagnosis of PMR (which may have been masked to some extent by the glucocorticoid treatment) or the development of co-morbidities with onset during PMR treatment (related or unrelated to PMR), having an impact of the functioning of the individual when glucocorticoid treatment is reduced.

The most recent guidance from the EULAR and ACR [3] recommends consideration of specialist referral in patients experiencing or at high risk of therapy-related side-effects, PMR refractory to glucocorticoid therapy and/or relapses/prolonged therapy. It is not yet understood how these patients at higher risk can be identified to facilitate early specialist referral.

The PMR Cohort study is an inception cohort of UK primary care patients diagnosed with PMR between 2012 and 2014 and initially followed up by mail over a 2-year period [14–16]. In this paper, we report on a further follow-up, between 4.5 and 6.5 years after the initial PMR diagnosis, in which we describe the characteristics of those with prolonged treatment and try to understand the reasons for this. Factors considered include the heterogeneity of the disease group, the co-morbidity profile, the potential role of glucocorticoid treatment patterns, and the initial trajectory of pain and stiffness symptoms after diagnosis [14].

## Methods

### PMR cohort study

**Fig. 1:**
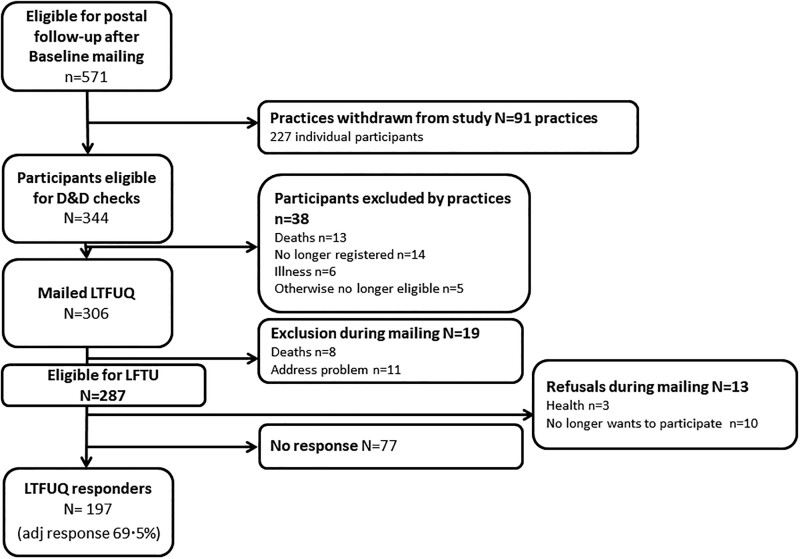
Participant flow chart for the long-term follow-up study

Full details of the PMR Cohort study have been published previously [14–16]. Briefly, 652 individuals were recruited from general practices across England at the time of their PMR diagnosis (2012–14). They completed postal surveys at diagnosis and after 1, 4, 8, 12, 18 and 24 months. Of the 571 individuals who had not withdrawn from the study or died by the time of the 24-month follow-up, 306 (53.6%) were still alive and registered with practices that agreed to participate in a further follow-up in 2019 (Fig. 1). These individuals were mailed the long-term follow-up (LTFU) questionnaire requesting information regarding their current PMR symptoms and treatment and their overall health. Those who did not respond were sent postal reminders after 2 and 4 weeks.

### Data collected

At each wave of the survey, participants reported their current levels of PMR-related pain and stiffness on numerical rating scales (scored 0–10) and the locations of their pain and stiffness on body manikins. Physical functioning was assessed by the modified Health Assessment Questionnaire (mHAQ) [17, 18]. Current prednisolone dose was recorded at each data collection point, and information on other medications was collected at baseline. Participants were also asked to report on their general health (EuroQol (EQ5D) [19]), mental health (Patient Health Questionnaire (PHQ8) [20] and Generalized Anxiety Disorders (GAD7) [21]), sleep (insomnia severity index [22]) and fatigue levels (Functional Assessment of Chronic Illness Therapy – Fatigue Scale (FACIT-fatigue) [23]). The LTFU questionnaire requested additional information regarding glucocorticoid treatment over time, co-morbidities and when they were diagnosed, other relevant health problems, symptom flares and weight changes.

### Sample size

The justification for the sample size recruited to this cohort has been described previously [15] and was based on the likely numbers of people diagnosed with PMR in UK primary care each year. The present study presents data from an additional follow-up, in which all those who had not withdrawn their consent and whose general practice agreed to continue participation were invited to continue to complete a final questionnaire.

### Statistical analyses

Responders to the LTFU survey were compared with the overall baseline sample and with the responders at 24-month follow-up using simple descriptive statistics to understand the extent of any attrition bias. Owing to the number of comparisons made, hypothesis testing was not used.

Responders to the LTFU who reported themselves to be still taking or to have stopped treatment were compared in terms of their glucocorticoid exposure over time, their baseline characteristics and changes in PMR symptoms between baseline and 1-, 4- and 24-months follow-up using Kruskall–Wallis, χ^2^ and 2-sample Student’s *t*-tests as appropriate.

Changes in key PMR symptoms and other health characteristics between baseline and 1 month and between baseline and 4 months were compared between those who had and had not stopped treatment. Adjusted mean scores or probability of the outcomes were calculated as appropriate.

Adjustment to the association between participant characteristics and continued treatment for other variables was not made, because our interest was in association and not potential causation.

Responders to the baseline survey have previously been allocated to one of five classes based on the dual trajectory of their pain and stiffness over the first 2 years after their PMR diagnosis (1: sustained symptoms; 2: partial recovery, sustained moderate symptoms; 3: recovery before worsening; 4: rapid and sustained recovery; and 5: slow and continuous recovery) [14]. Continued use of glucocorticoids was also compared across these classes.

### Content analysis

Participants were asked whether they had ever changed their prednisolone dose without going to see their doctor and, if so, how? This free-text question was analysed using content analysis [24], whereby each response was read repeatedly by one author (B.S.S.) to achieve data immersion, then classified according to the overall meaning of the comments. Codes that were closely related were combined into broader categories. A second author (S.M.) then repeated the process, and any disagreements were resolved by discussion. This process was carried out in Microsoft Excel. Ethical approval for the study was received from the Staffordshire Research Ethics Committee (REC reference number: 12/WM/0021), and all patients provided written informed consent.

### Patient and public involvement

Patients were involved in the design of study materials for all stages of the mailing process. A discussion was held with members of the Health Unlocked PMRGCAuk forum to understand aspects of the PMR diagnosis and treatment experience that patients felt were important to how their condition progressed.

## Results

### Cohort attrition

One hundred and ninety-seven responses to the LTFU were received (68.6% of those eligible). Those eligible to receive the LTFU questionnaire were broadly similar to the overall baseline sample (Supplementary Table S1, available at *Rheumatology Advances in Practice* online). Likewise, groups eligible and ineligible to receive the LTFU questionnaire after the 2-year follow-up were broadly similar in terms of their characteristics at baseline. At 2 years, however, the group eligible for the LTFU reported slightly higher pain and stiffness scores, and a higher proportion reported morning stiffness of >60 min and lived in less deprived areas. In those eligible for the LTFU, responders and non-responders were broadly similar in terms of their sociodemographic and PMR characteristics at baseline and 2 years, with the exception that responders lived in less deprived areas. Participants completed the LTFU questionnaire a median (quartile 1, quartile 3) of 5.16 (4.87, 5.57) years after their PMR diagnosis, with the longest time since diagnosis being 6.89 years.

### Prolonged treatment for PMR

Of the 179 people who reported ever taking glucocorticoid treatment for their PMR, 67 (40.1%) reported that they were still taking this treatment, with a median (quartile 1, quartile 3) daily dose of 5 (1.5, 9) mg (Table 1). Those who had stopped treatment reported having taken glucocorticoids for a total [mean (s.d.)] of 20.1 (13.8) months.

**Table 1:** Association of glucocorticoid treatment experience and baseline characteristics of continued glucocorticoid treatment at long-term follow-up

		PMR still treated, *n*/*N* (%)	PMR treatment stopped, *n*/*N* (%)	Missing, *n*/*N* (%)
		67/167 (40.1)	100/167 (59.9)	27/197 (13.7)
**Glucocorticoid treatment experience**				
Total time on glucocorticoid treatment, mean (s.d.), months^a^		N/A	20.1 (13.8)	22/100 (24.0)
Current glucocorticoid dose, median (Q1, Q3), mg		5 (1.5, 9)	N/A	7/67 (10.5)
Highest ever glucocorticoid dose, mean (s.d.), mg		20 (10, 20)	20 (15, 30)	65/179 (36.3)
Ever changed dose of own accord, *n* (%)		25/64 (39.1)	11/100 (11.0)	6/179 (3.4)
**Baseline characteristics**				
Sociodemographics and lifestyle				
Age, mean (s.d.), years		72.5 (6.8)	70.2 (8.5)	0
Gender, *n* (%)				
	Female	44/67 (65.7)	62/100 (62.0)	0
	Male	23/67 (34.3)	38/100 (38.0)	
IMD rank tertile, *n* (%)				5/179 (2.8)
	Most deprived	16/66 (24.2)	23/97 (23.7)	
	Middle	28/66 (42.4)	37/97 (38.1)	
	Least deprived	22/66 (33.3)	37/97 (38.1)	
Occupational class, *n* (%)				50/179 (27.9)
	Higher managerial, administrative and professional	21/53 (39.6)	18/70 (25.7)	
	Intermediate	13/53 (24.5)	27/70 (38.6)	
	Routine and manual	19/53 (36.9)	25/70 (35.7)	
Living alone, *n* (%)				1/179 (0.6)
	Yes	21/66 (31.8)	15/100 (15.0)	
	No	45/66 (68.2)	85/100 (85.0)	
BMI category, *n* (%)				5/179 (2.8)
	≤24.9 kg/m^2^	21/63 (33.3)	36/99 (36.4)	
	25–29.9 kg/m^2^	24/63 (38.1)	40/99 (47.5)	
	≥30 kg/m^2^	18/99 (28.6)	16/99 (16.2)	
Never smoked, *n* (%)		40/66 (60.6)	59/99 (59.6)	3/179 (1.7)
Alcohol drinking frequency, *n* (%)				1/179 (0.6)
	Daily/almost daily	7/66 (10.6)	21/100 (21.0)	
	3 or 4 times a week	10/66 (15.2)	11/100 (11.0)	
	Once or twice a week to 1–3 times a month	22/66 (33.3)	30/100 (30.0)	
	Special occasions only/never	27/66 (40.9)	38/100 (38.0)	
PMR symptoms				
Pain severity (0–10 NRS; higher score = more intense pain), median (Q1, Q3)		8 (7, 10)	8 (7, 9)	1/179 (0.6)
Stiffness severity (0–10 NRS; higher score = worse stiffness), median (Q1, Q3)		8 (7, 9)	8 (6, 9)	0
Time of day when stiffness is experienced, *n* (%)				0
	None	(6.0)^*^	0	
	Morning	65/67 (97.0)	98/100 (98.0)	
	Lunchtime	44/67 (65.7)	54/100 (54.0)	
	Afternoon	43/67 (64.2)	50/100 (50.0)	
	Early evening	47/67 (70.2)	60/100 (60.0)	
	Late evening	54/67 (80.6)	71/100 (71.0)	
	During the night	51/67 (76.1)	77/100 (77.0)	
Duration of early morning stiffness, *n* (%)				1 (0.6)
	<1 h	14/67 (20.9)	34/99 (34.3)	
	>1 h	53/67 (79.1)	65/99 (65.7)	
Able to raise arms above head when first diagnosed, *n* (%)				1 (0.6)
	Yes	22/67 (32.8)	25/99 (25.3)	
	No	43/67 (64.2)	67/99 (67.7)	
	Don’t know	(3.0)^*^	7/99 (7.1)	
Duration of symptoms before initial consultation				1 (0.6)
	<1 week	(6.0)^*^	5 (5.0)	
	1–2 weeks	14 (20.9)	14 (14.4)	
	2–4 weeks	15 (22.4)	24 (24.2)	
	≥4 weeks	34 (57.8)	56 (56.6)	
Treatment and care				
Visits to doctor before PMR diagnosis, *n* (%)				4/179 (2.2)
	1	25/65 (38.5)	33/99 (33.3)	
	2	22/65 (33.9)	31/99 (31.3)	
	3	9/65 (13.4)	17/99 (17.2)	
	≥4	9/65 (13.4)	18/99 (18.2)	
Medicines taken for PMR (other than glucocorticoids), *n* (%)				
	Paracetamol	30/67 (44.8)	37/100 (37.0)	0
	Paracetamol and codeine	18/67 (26.9)	24/100 (24.0)	0
	NSAIDs	9/67 (13.4)	12/100 (12.0)	0
	Strong prescription painkillers	6/67 (9.0)	9/100 (9.0)	0
	Proton pump inhibitors	40/67 (59.7)	46/100 (46.0)	0
	Calcium and vitamin D	31/67 (46.3)	46/100 (46.0)	0
	Bone protection	18/67 (26.9)	28/100 (28.0)	0
	Antidepressants	(4.5)^*^	7/100 (7.0)	0
General health				
EQ5D score (higher score = better quality of life), mean (s.d.)		0.76 (0.62, 0.93)	0.80 (0.69, 1.00)	13/179 (7.3)
Fallen in the last 12 months, *n* (%)		16/64 (25.0)	21/99 (21.2)	4/179 (2.2)
Experience of other symptoms, *n* (%)				
	GCA^b^	33/67 (49.3)	38/100 (38.0)	0
	Swollen joints	19/67 (28.4)	32/100 (32.0)	0
	Systemic^c^	25/67 (37.3)	31/100 (31.0)	0
mHAQ score (higher score = poorer function), mean (s.d.)		0.43 (0.06, 1.19)	0.38 (0.00, 0.75)	7/179 (3.9)
FACIT-fatigue score (higher score = less fatigue), mean (s.d.)		36.5 (26.0, 44.1)	40.0 (27.1, 44.1)	5/179 (2.8)
Insomnia severity index category, *n* (%)				6/179 (3.4)
	No clinically significant/subthreshold insomnia	52/66 (78.8)	79/98 (80.6)	
	Moderate/severe insomnia	14/66(21.2)	19/98 (19.4)	
PHQ8 category, *n* (%)				10/179 (5.6)
	None/mild depression	49/64 (76.6)	76/94 (80.9)	
	Moderate/severe depression	15/64 (23.4)	18/4 (19.2)	
GAD7 category, *n* (%)				11/179 (6.2)
	None/mild anxiety	56/62 (90.3)	87/95 (91.6)	
	Moderate/severe anxiety	6/62 (9.7)	8/95 (8.4)	

a Excluding those who reported a treatment duration of ≥80 months in line with study mailing time line.

b Sudden headache, tender scalp, problems with vision, jaw pain.

c Fever, loss of appetite, weight loss.

* Value suppressed owing to cell count < 5.

N/A: not applicable; NRS: numerical rating scale; Q1: quartile 1; Q3: quartile 3 IMD: Indices of Multiple Deprivation; EQ5D: EuroQoL; FACIT: fatigue Functional Assessment of Chronic Illness Therapy – Fatigue Scale; PHQ: Patient Health Questionnaire; GAD: Generalised Anxiety Disorder; ISI: Insomnia Severity Index; mHAQ: modified Health Assessment Questionnaire.

### Treatment characteristics over time

There was little difference in the highest ever reported dose of prednisolone between those who had stopped and who were still receiving glucocorticoid treatment, but those who were still receiving treatment were significantly more likely to report having changed their dose without consulting their doctor (39.1 *vs* 11.0%, *P* < 0.0001). From content analysis of the 40 responses to the open question, we found that of those describing the changes to their dose (*n* = 28), six described having agreed to self-management with their doctor (three specifically to reduce the dose, three to manage more broadly), 11 described reducing their own dose (one faster and three slower than advised; three reported a specific reduction regimen), two discontinued their treatment, two people described increasing their dose and seven reported returning to a previously effective dose. Numbers were too small to consider whether the way in which participants self-managed their glucocorticoid treatment was associated with continued long-term treatment. There were no significant differences in the age and gender of those responding to the open question and those not responding.

### Differences in baseline characteristics between those continuing and ceasing treatment

Those still taking glucocorticoids were older (72.5 *vs* 70.2 years at baseline, *P* = 0.035; Table 1) and more likely to live alone (31.8 *vs* 15.0%, *P* = 0.01). There were no other significant differences in reported care for PMR, co-morbidities, function or other health constructs between those still receiving and those who had ceased treatment.

### Changes in PMR symptoms and general health over time in those continuing and ceasing treatment

There was a significant association between class membership (defined by pain and stiffness trajectory over the first 2 years of the study [14]) and glucocorticoid treatment at LTFU (Table 2). Those still on treatment were more likely to belong to pain–stiffness trajectory classes 2 (partial recovery, sustained moderate symptoms) and 3 (recovery before worsening) and less likely to belong to classes 4 (rapid and sustained recovery) or 5 (slow and continuous recovery).

**Table 2:** Changes in PMR symptoms and general health between baseline and 1 and 4 months with and without continued glucocorticoid treatment at long-term follow-up

	PMR still treated, *n*/*N* (%)	PMR treatment stopped, *n*/*N* (%)	Missing, *n*/*N* (%)
	67/167 (40.1)	100/167 (59.9)	27/197 (13.7)
Pain and stiffness class membership over 2 years, *n*/*N* (%)			0
1: sustained symptoms	(1.5)^*^	(1.0)^*^	
2: partial recovery, sustained moderate symptoms	23/67 (34.3)	19/100 (19.0)	
3: recovery before worsening	17/67 (25.4)	11/100 (11.0)	
4: rapid and sustained recovery	15/67 (22.4)	46/100 (46.0)	
5: slow and continuous recovery	11/67 (16.4)	23/100 (23.0)	
**1 month**			
PMR symptoms			
Pain severity (0–10 NRS; higher score = more intense pain), adjusted mean (95% CI)	1.59 (1.55, 1.63)	1.60 (1.57, 1.63)	4/179 (2.2)
Stiffness severity (0–10 NRS; higher score = worse stiffness), adjusted mean (95% CI)	1.66 (1.63. 1.70)	1.65 (1.63. 1.67)	4/179 (2.2)
Time of day when stiffness is experienced, adjusted percentage (95% CI)^a^			
None	23.2 (22.6, 23.8)	31.6	3/179 (1.7)
Morning	64.1	62.5	3/179 (1.7)
Lunchtime	13.9 (12.7, 15.0)	14.9 (13.6, 16.2)	3/179 (1.7)
Afternoon	20.9	13.9 (13.1, 14.6)	3/179 (1.7)
Early evening	27.7	18.7 (18.0, 19.3)	3/179 (1.7)
Late evening	34.0 (33.0, 34.9)	22.9 (21.9, 24.0)	3/179 91.7)
During the night	16.9 (16.0, 17.7)	20.3 (19.0, 21.6)	3/179 (1.7)
General health			
mHAQ score (higher score = poorer function), adjusted mean (95% CI)	0.28 (0.26, 0.30)	0.29 (0.28, 0.31)	4/179 (2.2)
FACIT-fatigue score (higher score = less fatigue), adjusted mean (95% CI)	41.53 (41.07, 42.00)	41.28 (40.90, 41.66)	4/179 (2.2)
ISI moderate/severe insomnia, adjusted probability (95% CI)	36.7 (34.7, 38.8)	39.0 (35.7, 42.3)	6/179 (3.4)
PHQ8 moderate/severe depression, adjusted probability (95% CI)	12.1 (9.80 14.4)	11.0 (8.9, 13.0)	14/179 (7.8)
GAD7 moderate/severe anxiety, adjusted probability (95% CI)	9.4 (6.6, 12.1)	12.7 (10.6, 14.8)	11/179 (6.1)
**4 months**			
PMR symptoms			
Pain severity (0–10 NRS; higher score = more intense pain), adjusted mean (95% CI)	2.00 (1.97, 2.03)	2.01 (1.99, 2.04)	5/179 (2.8)
Stiffness severity (0–10 NRS; higher score = worse stiffness), adjusted mean (95% CI)	2.26 (2.07, 2.45)	2.35 (2.22. 2.48)	6/179 (3.4)
Time of day when stiffness is experienced, *n* (%)			
None	0.26	0.23	4/179 (2.2)
Morning	72.4 (71.8, 72.9)	0.67	6/179 (3.4)
Lunchtime	11.1 (10.8. 11.4)	21.4 (20.5, 22.4)	6/179 (3.4)
Afternoon	16.4 (16.0, 16.8)	25.3 (23.6, 27.0)	6/179 (3.4)
Early evening	25.6 (25.1, 26.2)	23.6 (22.4, 24.9)	6/179 (3.4)
Late evening	34.5 (33.5, 35.5)	34.8 (32.7, 36.9)	6/179 (3.4)
During the night	17.2 (16.3, 18.0)	28.0 (27.3, 28.7)	6/179 (3.4)
General health			
mHAQ score (higher score = poorer function), adjusted mean (95% CI)	0.39 (0.36, 0.43)	0.37 (0.34, 0.39)	4/179 (2.2)

a Where no CI is given, the value is the raw percentage, because the model was not adjusted for the baseline value owing to perfect prediction.

* Value suppressed owing to cell count < 5.

NRS: numerical rating scale.

There were no significant differences in baseline-adjusted PMR symptoms or general health measures at 1-month follow-up between those who had and had not ceased treatment at the LTFU. At the 4-month follow-up, there were no baseline-adjusted differences in pain or stiffness severity or in physical function. The baseline-adjusted probability of reporting stiffness at lunchtime and in the afternoon was lower in those still on treatment at the LTFU than in those who had stopped treatment.

### Co-morbidities and medication use

There was no significant difference in any specific self-reported co-morbidity or the number of co-morbidities reported on the LTFU questionnaire, either ever or with onset during the study period (Supplementary Table S2 available at *Rheumatology Advances in Practice* online). Those still receiving glucocorticoid treatment at the LTFU were more likely to be treated with proton pump inhibitors (65.7 *vs* 41.0%, *P* = 0.002), calcium and vitamin D (67.2 *vs* 27.0%, *P* < 0.001) and bone prophylaxis (e.g. bisphosphonates) (31.3 *vs* 8.0%, *P* < 0.001) at the LTFU than those who had ceased glucocorticoids. There were no other differences between those still receiving and having stopped treatment either at baseline or at the LTFU (Table 3).

**Table 3:** Self-reported medications at baseline

Medication	Current at long-term follow-up years, *n* (%)		Before PMR diagnosis, *n* (%)	
	PMR still treated [67 (40.1)]	PMR treatment stopped [100 (59.9)]	PMR still treated [67 (40.1)]	PMR treatment stopped [100 (59.9)]
Paracetamol	25/67 (37.3)	45/100 (45.0)	19/67 (28.4)	36/100 (36.0)
Paracetamol and codeine	11/67 (16.4)	17/100 (17.0)	6/67 (9.0)	11/100 (11.0)
NSAIDs	(6.0)^*^	12/100 (12.0)	7/67 (10.5)	17/100 (17.0)
Strong prescription painkillers	6/67 (9.0)	12/100 (12.0)	(6.0)^*^	8/100 (8.0)
Proton pump inhibitors	44/67 (65.7)	41/100 (41.0)	12/67 (17.9)	17/100 (17.0)
Calcium and vitamin D	45/67 (67.2)	27/100 (27.0)	(6.0)^*^	11/100 (11.0)
Bone protection	21/67 (31.3)	8/100 (8.0)	(1.5)^*^	7/100 (7.0)
AZA	0	0	0	0
MTX	(3.0)^*^	(2.0)^*^	(1.5)^*^	0
Anti-anxiety drugs/antidepressants	5/67 (7.5)	10/100 (10.0)	(4.5)^*^	10/100 (10.0)

Participants could report using a medication in both time periods. Participants were not asked to specify that this was for their PMR.

* Value suppressed owing to cell count < 5.

## Discussion

In keeping with some recent studies [10, 11], 40% of people in the present study reported using glucocorticoid treatment at a median follow-up time of >5 years after their initial PMR diagnosis. Despite having collected detailed data on this cohort, we were unable to find associations either at baseline or early follow-ups to provide insight into those more likely to require prolonged treatment. People continuing treatment were older at diagnosis and more likely to live alone. They were also more likely to have changed their glucocorticoid dose without consulting their doctor, but it is unclear whether this was a cause of prolonged treatment or a result of difficulties in dose reduction.

The only clear indicator of long-term treatment was the trajectory of pain and stiffness over the first 2 years of the condition. This is unlikely to aid in understanding prognosis at an early enough stage to suggest who needs specialist referral for glucocorticoid-sparing treatment. It might, however, be possible for general practitioners (GPs) to identify people within this 2-year period who report sustained high levels of symptoms and to enhance the monitoring of these patients.

The finding of prolonged treatment for 40% of people is congruent with the findings from an American cohort [10], in which the median time to treatment discontinuation was 5.95 years, although it is higher than the estimated 25% still using glucocorticoids 5 years after diagnosis in a recent meta-analysis [12]. In the UK Clinical Practice Research Datalink, the median time for continuous treatment after diagnosis (defined as <90 days between prescriptions) was reported to be 15.8 months, with 25% receiving treatment for >4 years in total [11]. In the present study, we do not know whether treatment was stopped and restarted between follow-ups. Regardless of the cause and effect of this prolonged treatment, it has been shown to be of concern to patients [6, 7], and there is evidence for the development of iatrogenic morbidities, even at relatively low doses of glucocorticoid [25].

This study has numerous strengths, including recruitment and follow-up in real time from primary care. This will reduce recall bias (although some might remain in terms of duration of glucocorticoid treatment reported in the LTFU questionnaire, especially in those patients no longer taking glucocorticoids) and provides a more representative sample than might be the case in samples recruited from specialist care settings, given that the majority of patients in the UK are diagnosed and managed exclusively in general practice [26, 27]. Although it could be argued that the GP diagnosis of PMR might be less accurate than a diagnosis made by a rheumatologist, we did not exclude those with a diagnosis made by a specialist, and GPs were provided with diagnostic guidelines from the British Society for Rheumatology [4] to support accurate participant recruitment. The age and gender profile of the present cohort is similar to those reported in studies in specialist settings, and we have previously conducted sensitivity analyses, producing similar findings in a subgroup of the cohort meeting a stricter definition of PMR at baseline [14].

The level of loss to follow-up at the LTFU stage is to be expected from a longitudinal cohort of older people. A large contributor to study attrition was the withdrawal of GP practices from the study, often attributable to competing clinical pressures and changes in GP administrative arrangements. Although the smaller numbers will result in less stable statistical estimates, we think this is unlikely to result in substantial differences between responders and non-responders, because practice withdrawal was not related to the PMR status of patients. The number of early disease characteristics (at baseline, 1- and 4-month follow-ups) considered in the analysis, combined with the relatively small sample size, increases the chances of a type I error (i.e. a falsely significant finding). To counteract this possibility, we have avoided over-interpretation of the findings. We have also chosen not to attempt to fit formal prognosis models, which would risk being under-powered and of less use to clinicians.

Previous work in this cohort has suggested that there are potentially subtypes of PMR [14]. Although the present study has not enabled us to identify these subtypes at an earlier point in the disease course, the association of these classes of individuals, based on pain and stiffness scores over 2 years, with the continued use of glucocorticoids a median of 5 years after diagnosis provides further evidence of these subtypes. Cessation of treatment after 5 years was more likely in those considered to have rapid and sustained or slow and continuous recovery than those classified as partial recovery, sustained moderate symptoms or recovery before worsening. This strengthens the need for on-going and close monitoring of PMR patients over the long term and, as advised by guidance, referral for specialist review either in circumstances when symptoms persist (reflecting the group with sustained moderate symptoms) or if people are still taking glucocorticoids by 2 years [4]. However, referral might also be considered if patients fall into the classes described above, which are associated with prolonged glucocorticoid treatment.

Although there are some limitations to this study, including the self-reported nature of the data and lack of information on inflammatory markers, there is evidence that many chronic conditions are well reported by research participants [28, 29]. The notable exception to this is the ability of participants to self-report subtypes of arthritis, which might explain the high prevalence of reported RA [30]. Also, we did not have records on the concomitant presence of GCA, which often co-occurs with PMR and requires a higher starting dose of glucocorticoids but has previously been shown to have a shorter duration of treatment [31]. Nor were we able to investigate the effect of the participants’ general practice on continued glucocorticoid use, owing to the small numbers of participants recruited from each practice. However, we are not aware of any other cohorts as detailed, representative or large as the one in the present study in which to take forward a prognosis model for treatment cessation in PMR. If it were possible to set up a new study in which to attempt to fit such a model, it would expensive and time consuming. It is therefore unlikely that the PMR research community will be able to predict who requires early specialist referral for glucocorticoid-sparing treatment in the foreseeable future. With this in mind, and with clear evidence that glucocorticoid medication and its withdrawal are major concerns for patients [6, 7], clinicians should give careful consideration to how treatment and its potential side-effects are managed in PMR. This might be possible through structured regular reviews, perhaps with other members of the multidisciplinary team, such as nurses or pharmacists, to identify when moderate symptoms persist or when glucocorticoid reduction is not proceeding as expected [32]. Research should also work towards safe and cost-effective alternative and adjunct treatments.

## Supplementary Material

rkac034_Supplementary_Data
