# Supplementary material for: Long-term use of glucocorticoids for polymyalgia rheumatica: follow-up of the PMR Cohort Study
Source: Rheumatol Adv Pract. 2022 May 11;6(2):rkac034. doi: 10.1093/rap/rkac034 (PMC9113421; doi:10.1093/rap/rkac034)
Supplement: rkac034_Supplementary_Data [file rkac034_supplementary_data.docx]

**Table of contents**

Page 2 Supplementary Table S1: Patient Characteristics and Attrition at LTFU according to baseline characteristics (n/N(%) unless otherwise specified)

Page 6 Supplementary Table S2: Self-reported comorbidities

**Supplementary Table S1 Patient Characteristics and Attrition at LTFU according to baseline characteristics (n/N(%) unless otherwise specified)**

| **Baseline characteristic** | | **Overall baseline sample (n=652)** | **24-month responders not eligible for follow-up (n=286)** | **24-month responders eligible for follow-up (n=287)** | **LTFU responders (n=197)** |
| --- | --- | --- | --- | --- | --- |
| Age (years) (Mean (SD)) | | 72.4 (9.3) | 72.4 (9.9) | 71.8 (8.6) | 72.0 (8.2) |
| Gender | |  |  |  |  |
|  | Male  Female | 247/652 (37.9)  405/652 (62.1) | 107/286 (37.4)  179/286 (62.6) | 110/287 (38.3)  177/287 (61.7) | 72/197 (36.6)  125/197 (63.5) |
| IMD rank | |  |  |  |  |
|  | Most deprived third  Middle third  Least deprived third | 208/633 (32.9)  215/633 (34.0)  210/633 (33.2) | 96/279 (34.4) 98/279 (35.1)  85/279 (30.5) | 80/199 (28.8)  92/199 (33.1)  106/199 (38.1) | 50/191 (26.2) 72 /191 (37.7) 69/191 (36.1) |
| Occupational class | |  |  |  |  |
|  | Higher managerial, admin & professional  Intermediate  Routine & manual | 144/447 (32.2)  125/447 (28.0)  178/447 (39.8) | 70/198 (35.4)  57/198 (28.8)  71/198 (35.9) | 64/286 (32.2)  57/286 (28.6)  78/286 (39.2) | 47/143 (32.9)  43/143 (30.1)  53/143 (37.1) |
| Ethnicity | |  |  |  |  |
|  | White  Non-white | 638/650 (98.2)  12 /650(1.8) | 280/285 (98.3)  5/285 (4.8) | 280/286 (97.9)  6/286 (2.1) | 192/196 (98.0)  *(2.0) |
| Employment status (n (%)) | |  |  |  |  |
|  | Employed  Retired  Other | 76/645 (11.8)  512/645 (79.4)  57/645 (8.8) | 34/285 (11.9)  228/285 (80.0)  23/285 (8.1) | 39/282 (13.8)  216/282 (76.6)  27//282 (9.6) | 24/193 (12.4)  154/193 (79.8)  15/193 (7.8) |
| Marital status | |  |  |  |  |
|  | Married  Widowed  Other | 411/648 (63.4)  143/648 (22.1)  94/648 (14.5) | 171/284 (60.2)  68/284 (23.9)  45/284 (15.9) | 192/286 (67.1)  57/286 (19.9)  37/286 (12.9) | 140/196 (71.4)  38/196 (19.4)  18/196 (9.2) |
| Pain (0-10 NRS) (Median (Q1, Q3)) | | 8 (7, 9) | 8/ (7, 9) | 8 (7,9) | 8 (7, 9) |
| Stiffness (0-10 NRS) (Median (Q1, Q3)) | | 8 (7, 9) | 8 (7, 9) | 8 (6.8, 9) | 8 (7, 9) |
| Morning stiffness duration | |  |  |  |  |
|  | ≤60 minutes  >60 minutes | 184/636 (28.9)  452/636 (71.1) | 83/273 (30.4)  190/273 (69.6) | 74/285 (26.0)  211/285 (74.0) | 55/196 (28.1)  14/196 (71.9) |
| Currently taking prednisolone | |  |  |  |  |
|  | No  Yes | 19/644 (3.0)  625/644 (97.1) | 11/284 (3.9)  273/284 (96.1) | *(1.4)  278/282 (98.6) | *(1.0)  194/196 (99.0) |
| PHQ8 score | |  |  |  |  |
| None/mild depression  Moderate/severe depression | | 470/601 (78.2)  131/601 (21.8) | 212/259 (81.9)  47/259 (18.2) | 204/269 (75.8)  65/269 (24.2) | 145/184 (78.8)  39/184 (21.2) |
| GAD7 score | |  |  |  |  |
|  | None/mild anxiety  Moderate/severe anxiety | 530/609 (87.0)  79/609 (13.0) | 232/265 (87.6)  33/265 (12.5) | 235/267 (88.0)  32/267 (12.0) | 168/184 (91.3)  16/194 (8.7) |
| mHAQ score (Median (Q1, Q3)) (higher score=poorer physical function) | | 0.40 (0.00, 1.00) | 0.38 (0.00, 0.88) | 0.38 (0, 0.88) | 0.38 (0.00, 0.88) |
| EQ5D score (Median (Q1, Q3)) (higher score=better quality of life) | | 0.73 (0.59, 0.85) | 0.73 (0.59, 0.85) | 0.76 (0.62, 0.86) | 0.76 (0.66, 0.88) |
| FACIT-Fatigue (Median (Q1, Q3)) (higher score=less fatigue) | | 36.6 (24.0, 44.0) | 37.4 (26.0, 44.3) | 37.0 (26.0, 44.0) | 38.5 (27.0, 44.0) |
| ISI score | |  |  |  |  |
|  | No clinically significant/subthreshold insomnia  Moderate/severe clinical insomnia | 469/614 (76.4)  145/614 (23.6) | 203/262 (77.5)  59/262 (22.5) | 217/279 (77.8)  62/279 (22.2) | 153/190 (80.5)  37/190 (19.5) |
| Never smoked | | 317/643 (49.3) | 139/282 (49.3) | 152/284 (53.5) | 113/194 (58.3) |
| Alcohol drinking frequency | |  |  |  |  |
|  | Daily/almost daily  3 or 4 times a week  Once or twice a week - 1 to 3 times a month  Special occasions only / Never | 82/650 (12.6)  82/650 (12.6)  193/650 (29.7)  293/650 (45.1) | 34/285 (11.9)  33/285 (11.6)  87/285 (30.5)  131/285 (46.0) | 45/286 (15.7)  36/286 (12.6)  88/286 (30.8)  117/286 (40.9) | 32/196 (16.3)  27/196 (13.8)  58/196 (29.6)  79/196 (40.3) |
| BMI | |  |  |  |  |
|  | <24.9 kg/m^2^  25 to 29.9 kg/m^2^  ≥30 kg/m^2^ | 214/625 (34.2)  251/625 (40.2) 160/625 (25.6) | 91/271 (33.6)  108/271 (39.9)  72/271 (26.6) | 97/277 (35.0) 114/277 (41.2) 66/277 (23.8) | 65/191 (34.0)  83/191 (43.5) 43/191 (22.5) |
| Pain and stiffness class membership over 2 years | |  |  |  |  |
|  | 1 – Sustained symptoms  2 – Partial recovery, sustained moderate symptoms  3 – Recovery before worsening  4 – Rapid and sustained recovery  5 – Slow and continuous recovery | 52/652 (8.0)  157/652 (24.2)  106/652 (16.3)  224/652 (34.5)  111/652 (17.1) | 22/286 (7.7)  57/286 (20.0)  58/286 (20.4)  99/286 (34.7)  49/286 (17.2) | 15/287 (5.2)  75/287 (26.1)  43/287 (15.0)  99/287 (34.5)  55/287 (19.2) | 5/197 (2.5)  52/197 (26.4)  31/197 (15.7)  72/197 (36.6)  37/197 (18.8) |
| **24-month characteristics** | |  |  |  |  |
| Employment status | |  |  |  |  |
|  | Employed  Retired  Other | - | 23/205 (11.2)  160/205 (78.1)  22/205 (10.7) | 17/227 (7.5)  186/227 (81.9)  24/227 (10.6) | 17/177 (9.6)  141/177 (79.7)  19/177 (10.7) |
| Marital status | |  |  |  |  |
|  | Married  Widowed  Other | - | 130/210 (61.9)  45/210 (21.4)  35/210 (16.7) | 152/229 (66.4)  56/229 (24.5)  21/229 (9.2) | 121/180 (67.2)  42/180 (23.3)  17/180 (9.4) |
| Pain (0-10 NRS) (Median (Q1, Q3)) | | - | 2 (0, 5) | 3 (0, 5) | 3 (0, 5) |
| Stiffness (0-10 NRS) (Median (Q1, Q3)) | | - | 2 (1, 5) | 3 (1, 5) | 3 (1, 5) |
| Morning stiffness duration | |  |  |  |  |
|  | ≤60 minutes  >60 minutes | - | 161/207 (77.8)  46/207 (22.2) | 155/226 (68.6)  71/226 (31.4) | 120/176 (68.2)  56/176 (31.8) |
| Currently taking prednisolone | |  |  |  |  |
|  | No  Yes | - | 93/210 (44.3)  117/210 (55.7) | 89/227 (39.2)  138/227 (60.8) | 66/176 (37.5)  110/176 (62.5) |
| PHQ8 score | |  |  |  |  |
| None/mild depression  Moderate/severe depression | | - | 166/187 (88.8)  21/187 (11.2) | 187/216 (86.6)  29/216 (13.4) | 151/169 (89.4)  18/169 (10.7) |
| GAD7 score | |  |  |  |  |
|  | None/mild anxiety  Moderate/severe anxiety | - | 180/198 (90.9)  18/198 (9.1) | 202/220 (91.8)  18/220 (8.2) | 160/172 (93.0)  12/172 (7.0) |
| mHAQ score (Median (Q1, Q3)) (higher score=poorer physical function) | | - | 0.25 (0.00, 0.72) | 0.25 (0.00, 0.75) | 0.25 (0.00, 0.69) |
| EQ5D score (Median (Q1, Q3)) (higher score=better quality of life) | | - | 0.73 (0.59, 0.85) | 0.69 (0.62, 0.80) | 0.73 (0.62 ,0.80) |
| FACIT-Fatigue (Median (Q1, Q3)) (higher score=less fatigue) | | - | 39.5 (28.8, 45.1) | 40.0 (30.0, 46.0) | 40.2 (33.0, 46.0) |
| ISI score | |  |  |  |  |
|  | No clinically significant/subthreshold insomnia  Moderate/severe clinical insomnia | - | 181/203 (89.2)  22/203 (10.8) | 191/225 (84.9)  34/225 (15.1) | 152/177 (85.9)  25/177 (14.1) |
| BMI | |  |  |  |  |
|  | <24.9 kg/m^2^  25 to 29.9 kg/m^2^  ≥30 kg/m^2^ | - | 69/193 (35.8)  69/193 (35.8)  55/193 (28.5) | 72/220 (32.7)  97/220 (44.1) 51/220 (23.2) | 55/172 (32.0) 77/172 (44.8)  40/172 (23.3) |

Not eligible for LTFU mailing at time of mailing

SD – standard deviation; Q1 – quartile 1; Q3 – quartile 3; kg/m^2^ - kilograms per metre squared

*value suppressed due to cell count<5

**Supplementary Table S2: Self-reported comorbidities**

| **Comorbidity** | **Reported ever to have/have had at LTFU, n/N(%)** | | **Onset during course of PMR, n/N(%^1^)** | |
| --- | --- | --- | --- | --- |
|  | **PMR still treated (67 (40.1%))** | **PMR treatment stopped (100 (59.9%))** | **PMR still treated (67 (40.1%))** | **PMR treatment stopped (100 (59.9%))** |
| Number (median, IQR) | 3 (2, 4) | 3 (1, 4) | 1 (0, 3) | 1 (0, 2) |
| Gastrointestinal reflux disease | 30/50 (60.0) | 32/65 (47.8) | 16/30 (53.3) | 19/32 (59.4) |
| Diabetes mellitus | 13/40 (32.5) | 11/56 (19.0) | 9/13 (69.2) | *(36.4) |
| Giant cell arteritis | *(10.8) | *(7.3) | 0 | *(50.0) |
| Rheumatoid arthritis | 22/45 (48.9) | 24/56 (41.4) | 5/22 (22.7) | 11//24 (45.8) |
| Cancer | 12/42 (28.6) | 18/62 (28.1) | 5/12 (41.7) | 8/18(44.4) |
| Heart disease | 8/36 (22.2) | 14/56 (25.0) | 5/8 (62.5) | 10/14 (71.4) |
| Hypertension | 35/49 (71.4) | 45/70 (64.3) | 25/35 (71.4) | 36/45 (80.0) |
| Hypercholesterolemia | 23/45 (51.1) | 30/60 (50.0) | 18/23 (78.3) | 19/30 (61.3) |
| Osteoporosis | 23/46 (50.0) | 20/62 (31.3) | *(13.0) | 5/25 (25.0) |
| Stroke | 53/35 (14.3) | 6/55 (10.5) | *(60.0) | *(50.0) |
| Cataracts | 25/44 (56.8) | 30/62 (46.9) | 8/25 (32.0) | 12/30 (40.0) |
| Depression | 6/39 (15.4) | 19/58 (31.7) | *(50.0) | 12/19 (63.2) |
| Anxiety | 11/41 (26.8) | 18/55 (32.7) | *(36.4) | 9/18 (50.0) |

^1^As a percentage of all those reporting condition

*value suppressed due to cell count<5
